# *Brassica rapa* orphan genes largely affect soluble sugar metabolism

**DOI:** 10.1038/s41438-020-00403-z

**Published:** 2020-11-01

**Authors:** Mingliang Jiang, Zongxiang Zhan, Haiyan Li, Xiangshu Dong, Feng Cheng, Zhongyun Piao

**Affiliations:** 1 Molecular Biology of Vegetable Laboratory, College of Horticulture, Shenyang Agricultural University, Shenyang, 110866 China; 2 School of Agriculture, Yunnan University, Kunming, 650504 China; 3 Key Laboratory of Biology and Genetic Improvement of Horticultural Crops of the Ministry of Agriculture, Sino-Dutch Joint Laboratory of Horticultural Genomics, Institute of Vegetables and Flowers, Chinese Academy of Agricultural Sciences, Beijing, 100081 China

**Keywords:** Plant molecular biology, Molecular engineering in plants, Gene regulation, Metabolism

## Abstract

Orphan genes (*OG*s), which are genes unique to a specific taxon, play a vital role in primary metabolism. However, little is known about the functional significance of *Brassica rapa OG*s (*BrOG*s) that were identified in our previous study. To study their biological functions, we developed a *BrOG* overexpression (*BrOG*OE) mutant library of 43 genes in *Arabidopsis thaliana* and assessed the phenotypic variation of the plants. We found that 19 of the 43 *BrOG*OE mutants displayed a mutant phenotype and 42 showed a variable soluble sugar content. One mutant, *BrOG1*OE, with significantly elevated fructose, glucose, and total sugar contents but a reduced sucrose content, was selected for in-depth analysis. *BrOG1*OE showed reduced expression and activity of the *Arabidopsis* sucrose synthase gene (*AtSUS*); however, the activity of invertase was unchanged. In contrast, silencing of two copies of *BrOG1* in *B. rapa, BraA08002322* (*BrOG1A*) and *BraSca000221* (*BrOG1B*), by the use of an efficient CRISPR/Cas9 system of Chinese cabbage (*B. rapa* ssp. *campestris*) resulted in decreased fructose, glucose, and total soluble sugar contents because of the upregulation of *BrSUS1b*, *BrSUS3*, and, specifically, the *BrSUS5* gene in the edited *BrOG1* transgenic line. In addition, we observed increased sucrose content and SUS activity in the *BrOG1* mutants, with the activity of invertase remaining unchanged. Thus, *BrOG1* probably affected soluble sugar metabolism in a SUS-dependent manner. This is the first report investigating the function of *BrOG*s with respect to soluble sugar metabolism and reinforced the idea that *OG*s are a valuable resource for nutrient metabolism.

## Introduction

Orphan genes (*OG*s), genes that are restricted to a single lineage or species, show either nonsignificant or no sequence similarity with genes in other related species. *OG*s have been identified in many species or lineages, such as *Arabidopsis thaliana*^1^, *Brassica rapa*^2^, and *Citrus sinensis*^3^, with the continuing sequencing of several genomes. Although there is limited information on the functional significance of *OG*s in a particular species, they are known to play a role in primary metabolism, respond to biotic and abiotic stresses, and influence species-specific evolution and species-specific traits. A study reported that a drought-inducible *Vigna unguiculata* OG, *UP12_8740*, induced increased tolerance to osmotic stresses and soil drought, indicating the vital role played by *OG*s in specific environmental adaptation^4^. The *A. thaliana* OG *Qua Quine Starch* (*QQS*) is known to regulate the primary metabolic functions of carbon and nitrogen partitioning, and affected protein content when it was overexpressed in soybean, maize, and rice^5^. An in-depth analysis showed that *QQS* and its interactor *NF-YC4* could reduce *Arabidopsis* and soybean susceptibility to viruses, bacteria, fungi, aphids, and soybean cyst nematodes^6^. The salamander-specific protein *Prod1* can regulate limb regeneration by determining the direction of limb growth^7^. Currently, the prediction and characterization of the functions of most *OG*s are unclear, as they lack identifiable folds, recognizable domains, and functional motifs.

In plants, gene function can be investigated by employing a gain-of-function approach^8^. Target genes can be introduced into a plant genome under the control of a suitable constitutive promoter and terminator sequence to generate specific gain-of-function mutants and, subsequently, the overexpression of these genes can result in corresponding distinct phenotypes, which would help in deducing gene function^9^. A previous study showed that a full-length soybean cDNA overexpression mutant library in *A. thaliana*, which was constructed to study the function of soybean genes, provided an abundance of mutants for the genetic development of soybean^10^. Approximately 6000 *A. thaliana* transgenic lines were generated by overexpressing full-length *Brassica napus* cDNAs under the inducible FOX-Hunting system and several lines showed visible phenotypes post induction^11^. Therefore, the functional analysis of *B. rapa OG*s (*BrOG*s) could be performed rapidly based on the efficient transformation frequency and short generation time of *A. thaliana*. Until now, there have been no reports on the construction of a mutant library of *OG*s or on functional analysis of *BrOG*s.

*Brassica* vegetables are rich in nutrients and are planted and consumed worldwide^12^. These vegetables contain several types of soluble sugars, including sucrose (Suc) and its products glucose (Glc) and fructose (Fru), which provide energy and the basic carbon skeletons for various metabolic pathways^13,14^. There exist closes relationship between sugar signaling and developmental processes, and soluble sugars can act as signal transduction molecules to regulate development and adaptation to environmental challenges^15,16^. The hydrolysis products of Suc act as carbon and energy sources. Suc can be reversibly hydrolyzed by Suc synthase (SUS) to produce Fru and UDP-Glc, and irreversibly hydrolyzed by invertase (INV) to yield Fru and Glc^14,17^. There are six SUS isoforms in *Arabidopsis* that exhibit a high level of redundancy^18^. *Arabidopsis SUS* quadruple mutants (*sus1*/*sus2*/*sus3*/*sus4*) display normal growth and development, but the level of reduction in SUS activity varies among vegetative tissues^18,19^. There are several isoforms of acid INV, including acid INV isozymes in the cell wall (encoded by six genes, *CWINV*s) and vacuole (two genes, *VINV*), and neutral/alkaline INV isozymes (encoded by nine genes, *CINV*s) in the cytosol, plasma membrane, nucleus, mitochondrion, and chloroplast^18,20^. A study showed that mutations in *A. thaliana cinv1*/*cinv2* resulted in severe growth inhibition and abnormal cell division^18^. SUS plays a central role in photosynthetic carbon assimilation and partitioning, and INV is involved in specific developmental stages^18^; however, it is challenging to differentiate their in planta role^20^. Until now, there have been no reports on the relationships between *BrOG*s and soluble sugar metabolism. Thus, the identification of *BrOG*s that could influence soluble sugar metabolism in *B. rapa* could be substantially beneficial for improving the nutritional quality of this species.

Previous studies have used mutagenesis and genetic transformation to create new cultivars, which are known to facilitate breeding processes^21^. However, instead of using laborious selection strategies to remove random mutations introduced by traditional mutagenesis methods, recent studies have employed CRISPR/Cas9 (clustered regularly interspaced short palindromic repeat/CRISPR-associated Cas9) technology for targeted gene modification and precise gene editing in several organisms^22^. Several studies have reported successful CRISPR/Cas9-mediated mutations in many Cruciferae species, such as *A. thaliana*^23^, *Camelina sativa*^24^, *B. napus*^25^, and *Brassica oleracea*^26^, indicating its potential application in *B. rapa*, as well. Nonetheless, there are few reports on CRISPR/Cas9-induced mutations in transgenic *B. rapa* lines^27^. Therefore, an efficient gene-editing system is required to study the gene functions in *B. rapa*.

In a previous study, we identified *OG*s and revealed their possible roles in *B. rapa*^2^. However, their functional role remains unknown. Thus, we used *A. thaliana* as a host plant to perform a functional analysis of *BrOG*s. Here, we constructed a *BrOG* overexpression (BrOGOE) mutant library in *A. thaliana*, after which we characterized the phenotypic variations and the content of soluble sugars in the BrOGOE mutants. We performed an in-depth analysis of a specific *OG* (*BrOG1*) by establishing a gene-editing system, which revealed the potential role of *BrOG1* in soluble sugar metabolism. This is the first report presenting a comprehensive evaluation of the function of *BrOG*s involved in soluble sugar metabolism.

## Results

### Generation of a BrOGOE mutant library and phenotypic investigation

To explore the functional significance of *BrOG*s, we generated a BrOGOE mutant library in *A. thaliana*. We successfully transformed 43 *BrOG*s (gene IDs listed in Supplementary Table S1) into *A. thaliana* (Col-0) through floral-dip transformation with random selection. The CaMV 35S promoter controlled the expression of *BrOG*s. Efficient screening of transgenic plants was performed using the DsRed marker gene under the control of the CaMV 35S promoter, which resulted in the selection of T_2_ homozygous seeds from different self-pollinated T_1_ transgenic seed lines. The overexpression of the transgenes was confirmed by quantitative reverse-transcriptase PCR (qRT-PCR) for both the mutants and wild type (WT), and the results showed that the expression was detected only in the transgenic plants.

**Fig. 1:**
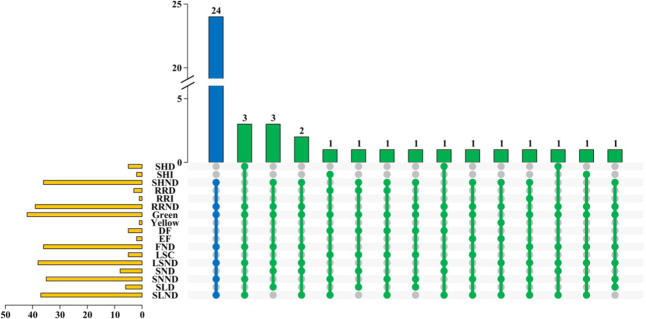
Characterization of different mutant types and phenotypic analysis of 43 BrOGOE mutants. The blue bar and the solid circle represent mutants whose phenotype did not differ compared with that of the wild type (WT). The green bar and the solid circle represent mutants with whose phenotype significant differed from that of the WT. The solid gray circle indicates the absence of a corresponding phenotype for that mutant. The yellow column in the lower-left corner represents the total number of mutants corresponding to the phenotype. SHND: stem height with no difference compared with that of the WT; SHD: decreased stem height; SHI: increased stem height; RRND: rosette radius, with no difference; RRD: decreased rosette radius; RRI: increased rosette radius (the leaf color is green and yellow); FND: flowering time, with no difference; DF: delayed flowering; EF: early flowering; LSND: leaf shape, with no difference; LSC: leaf shape change; SNND: seed number, with no difference; SND: decreased seed number; SLND: silique length, with no difference; SLD: decreased silique length

Next, we systematically investigated variations in the following phenotypic characteristics of the BrOGOE mutants using stable homozygous T_2_ transgenic plants during the vegetative and reproductive stages: stem height, rosette radius, leaf color, flowering time, leaf shape, seed number, and silique length. We found phenotypic variations in 19 BrOGOE mutants compared with the WT; 24 BrOGOE mutants showed no significant difference in phenotypes (Fig. 1 and Supplementary Table S1). *BrOG49*OE, *BrOG61*OE, and *BrOG91*OE displayed decreased-stem height phenotypes, *BrOG96*OE displayed increased-stem height phenotypes, *BrOG43*OE and *BrOG53*OE displayed decreased-seed number phenotypes, *BrOG111*OE showed a decreased-silique length phenotype, and *BrOG26*OE showed an increased-rosette radius phenotype (Fig. 1, Supplementary Table S1, and Supplementary Fig. S1). Interestingly, the remaining 11 BrOGOE mutants, such as *BrOG72*OE, showed at least two different phenotypes compared with that of the WT. Five and two BrOGOE mutants showed delayed-flowering and early-flowering phenotypes, respectively, and the phenotypes of these two mutants were shared with others.

### Screening for mutants in which the soluble sugar content is affected

**Fig. 2:**
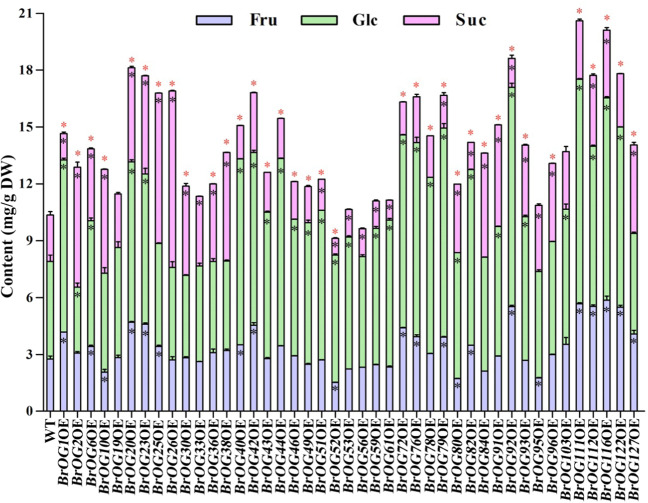
Variations in soluble sugar content in BrOGOE *Arabidopsis* lines. Wild-type Col-0 (WT) and BrOGOE plants were grown under LD conditions. Thirty-day-old plants were used to analyze the soluble sugars, including fructose (Fru, light blue bar), glucose (Glc, light green bar), and sucrose (Suc, light pink bar), simultaneously. The data in bar graphs are expressed as the means ± SEs, calculated from three replications. Each replicate consisted of an independent pool of 15 plants. The black asterisks inside the bars indicate significant differences between the mutants and WT for Fru, Glc, and Suc (*p* < 0.05) based on Student’s *t*-tests. The red asterisks at the top of the bars indicate significant differences in total soluble sugars between the mutants and WT (*p* < 0.05) based on Student’s *t*-tests

A previous study showed the significance of *OG*s in primary metabolism^5^. Soluble sugars are one of the main evaluation indicators of nutritional quality of *Brassica* vegetables^13^. Most of the mutants showed no distinct phenotype, indicating that these mutants were involved in primary metabolism. Thus, we screened the BrOGOE *Arabidopsis* lines to isolate mutants presenting variation in the contents of soluble sugars, such as Fru, Glc, and Suc. The soluble sugars were extracted from the shoots of representative 30-day-old T_2_ plants and measured through ultra-performance liquid chromatography (UPLC) (Fig. 2). After screening 43 mutant lines, we found that the Fru contents in 17 lines (such as *BrOG1*OE) was enhanced and those of 4 lines (such as *BrOG10*OE) decreased; 29 lines (such as *BrOG1*OE) showed a higher Glc content compared with that in the WT, whereas only one mutant (*BrOG2*OE) showed decreased Glc contents. Twenty lines (such as *BrOG2*OE) showed increased Suc contents and ten lines (such as *BrOG1*OE) showed decreased contents. In addition, 81.40% of the lines showed variations in total soluble sugar contents: 34 lines (such as *BrOG1*OE) showed increased contents, 1 (*BrOG52*OE) showed decreased contents, and only 8 lines (such as *BrOG19*OE) displayed no significant difference compared those in with WT. We observed a change in soluble sugar content in 42 *Arabidopsis* lines (*BrOG19*OE was excluded), which suggested that *BrOG*s could extensively impact soluble sugar metabolism.

### Sequence analysis of *BrOG1*

**Fig. 3:**
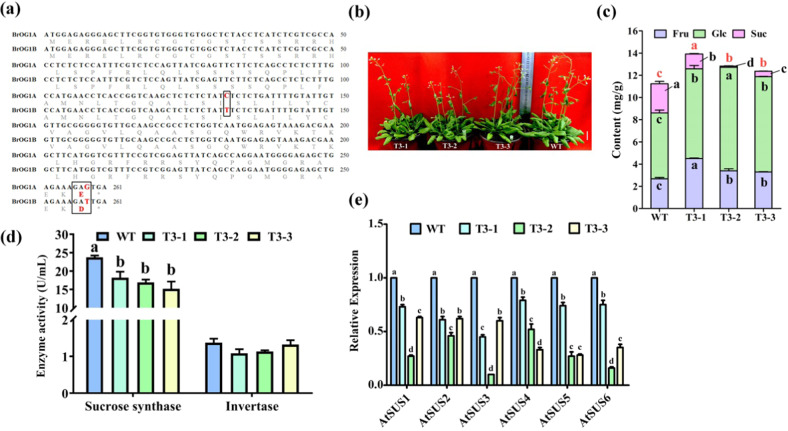
Characterization of *BrOG1A*OE mutants involved in soluble sugar metabolism in *A. thaliana*. **a** Sequence alignment of *BrOG*1A and *BrOG*1B. The coding sequences and protein sequences were obtained from the *Brassica* database (BRAD). The two solid black boxes represent synonymous mutations (132 bp, C to T) and nonsynonymous mutations (258 bp, G to T, causing E to D), respectively. **b** Phenotypes of *BrOG1A*OE T_3_ lines in *A. thaliana*. Representative images of 37-day-old mutants resulting from three independent genetic transformation events (T3-1, T3-2, and T3-3) and wild-type Col-0 (WT). The scale bars are 2 cm. **c** Soluble sugar content in 30-day-old wild-type (WT) and *BrOG1A*OE transgenic *Arabidopsis* plants. The analysis was performed in triplicate, with 15 plants per replicate. **d** Detection of enzyme activity of sucrose synthase (SUS) and invertase (INV). **e** Levels of *AtSUS* transcripts in WT and *BrOG1A*OE plants. **d**, **e** The analysis was performed in triplicate, with three plants per replicate. **c**–**e** All the values are expressed as the means ± SEs. The black letters indicate statistically different groups (one-way ANOVA, *p* < 0.05). **c** The red letters at the top of the bars indicate significant differences in total soluble sugars between the mutants and WT (one-way ANOVA, *p* < 0.05)

*BrOG1* (*BraA08002322*) was randomly chosen for preliminary analysis of its role in soluble sugar metabolism due to the high abundance of variation in soluble sugar contents in the BrOGOE library. *BrOG1* has a highly homologous gene located on the scaffold in the *B. rapa* reference genome (v2.5); in addition, *BrOG1* has a nucleotide sequence similarity of 99.23% and a protein sequence similarity of 98.84%. In this study, these two intronless genes were labeled *BrOG1A* (*BrOG1*) and *BrOG1B* (*BraSca000221*). We found sequence differences between *BrOG1A* and *BrOG1B* consisting of synonymous mutations (132 bp, C to T) and nonsynonymous mutations (258 bp, G to T, causing E to D) (Fig. 3a). Therefore, *BrOG1A* could represent the function of *BrOG1B* and was thus used to characterize the potential role of *BrOG1* in *Arabidopsis*. Protein sequence analysis of *BrOG*1A and *BrOG*1B showed that they had no conserved domains, signal peptides, or cleavage sites and were not identified as transcription factors.

### *BrOG1* probably influenced the content of soluble sugar in a SUS-dependent manner

We compared the phenotypic features between the *BrOG1A*OE mutant and WT during the vegetative and reproductive growth stages under LD conditions. The results showed a similarity between the phenotypes of the *BrOG1A*OE mutant and the WT (Supplementary Table S2 and Fig. 3b). Three T_3_ lines from independent genetic transformation events were further characterized for their soluble sugar content. Consistent with the results of T_2_ representative lines, overexpression of *BrOG1A* increased the contents of Fru, Glc, and total sugars, and decreased the contents of Suc compared with those in the WT (Fig. 3c). The activity of Suc-degrading enzymes, SUS and INV, were then analyzed in the *BrOG1A*OE mutant. There was no significant difference in AtINV activity of the *BrOG1A*OE mutant or the WT, whereas the AtSUS activity in the *BrOG1A*OE mutant significantly decreased compared with that in the WT (Fig. 3d). The decrease in AtSUS enzyme activity was further confirmed by expression analysis of all *AtSUS*s, which were found to be downregulated in the *BrOG1A*OE lines (Fig. 3e).

### Sequence validation of *BrOG1A* and *BrOG1B* in Chinese cabbage for genetic transformation

To confirm the potential role of *BrOG1* in Chinese cabbage, we knocked out the OG *BrOG1* in Chinese cabbage using CRISPR/Cas9-mediated mutation. Sequence identification of these two genes was performed in the self-propagating Chinese cabbage GT-24, which was also used for genetic transformation. A common specific primer pair (*BrOG*1-com F/R) was designed via upstream and downstream extension of these two genes. Sequence analysis of *BrOG1A* and *BrOG1B* in GT-24 and Chiifu revealed high similarity. These results suggested the occurrence of *BrOG1* gene-specific mutagenesis.

### Detection of sgRNA target activity and vector construction

**Fig. 4:**
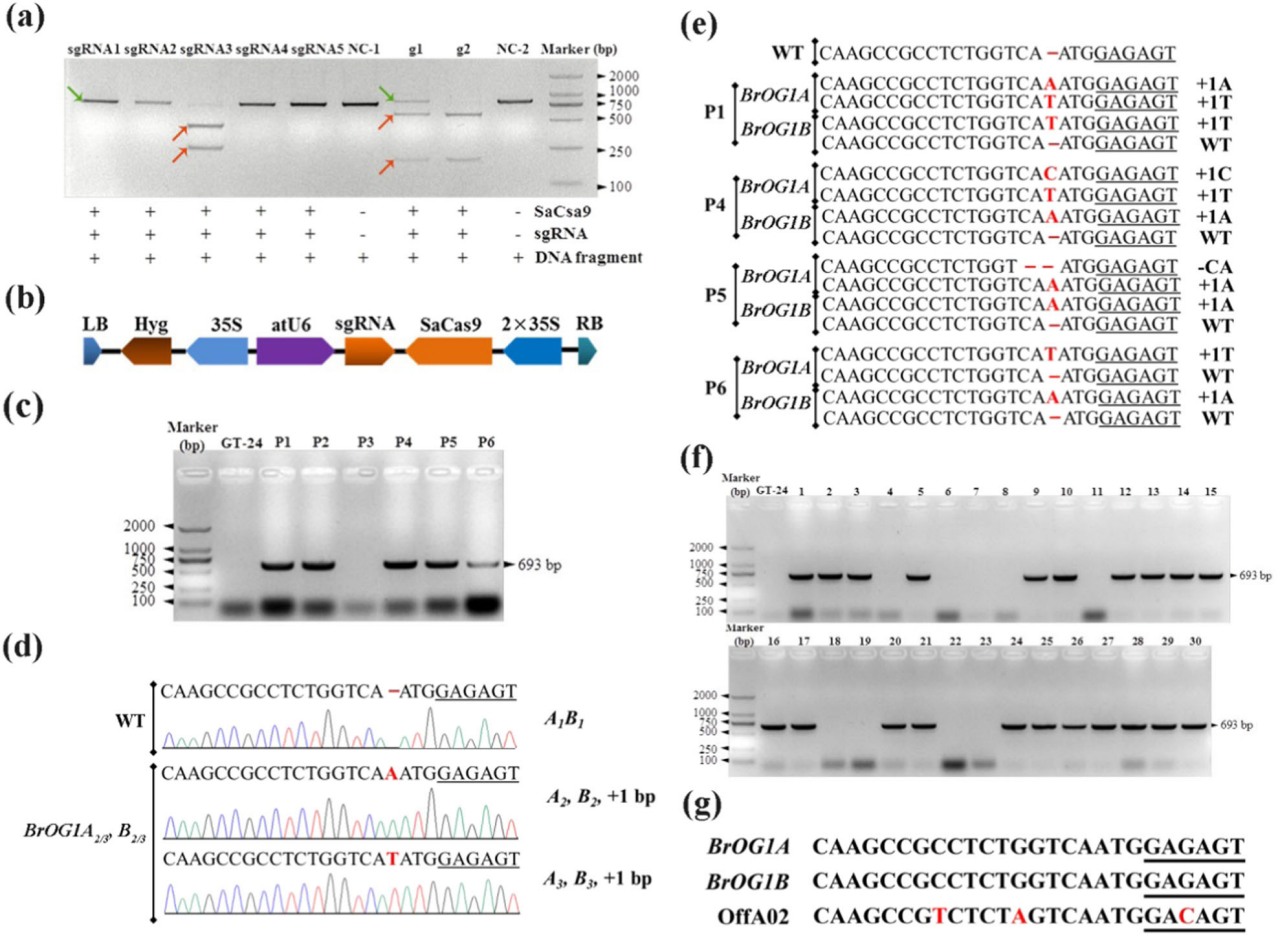
CRISPR/Cas9-mediated knockout of two *BrOG1* homeologs. **a** Detection of the target efficiency of sgRNAs in vitro. The DNA fragment indicates the in vitro transcription template of sgRNA (T7 promoter, target, and the sgRNA scaffold sequence). The red and green arrows indicate cut bands and uncut bands, respectively. The “+” and “−” symbols indicate that the corresponding substances were included and omitted, respectively. The negative controls NC-1 and NC-2 indicate the exclusive addition of the DNA fragments of sgRNA5 and g2, respectively. The g1 and g2 positive controls indicate standard gRNA1 and gRNA2, respectively. **b** Schematic diagram of the T-DNA region of the assembled SaCas9/sgRNA expression vector (VK005-101) for *Agrobacterium*-mediated transformation. RNA-guided *Staphylococcus aureus* Cas9 nuclease system (SaCas9) using both the *A. thaliana* ubiquitin 6-26 (atU6) promoter and a 2× 35S promoter was used to express a single guide RNA (sgRNA) scaffold. For plant selection, the constructs harbored a hygromycin (Hyg) resistance cassette. LB: left border, RB: right border. **c** Results of agarose gel electrophoresis for the detection of exogenous T-DNA insertion in six T_0_ plants. GT-24 indicates the nontransgenic control and P1–P6 indicate different lines in the T_0_ generation. Amplified bands of mutants carrying the transgene insertion occurred at 693 bp. **d** Four mutant alleles that were from one double heterozygous T_0_ plant (P2) generated by CRISPR/Cas9 were tested through Sanger sequencing. The name of the allele and the size of each insertion are shown on the right. The inserted base of the alleles is highlighted using the red color. WT indicates a GT-24 nontransgenic plant. The PAMs are underlined. **e** Sequencing of targeted mutations in transgenic T_0_ plants (P1, P4, P5, and P6). The size of each insertion and deletion of the alleles are indicated on the right; WT represents the absence of a mutation. **f** Detection of exogenous T-DNA insertions in T_1_ plants by PCR. Nos. 1–30 represent individual T_1_ plants from the inbreeding generations of line P2. **g** Alignment of the CRISPR/Cas9 target sequences from *BrOG1A* and *BrOG1B* compared to the sequence of a potential off-target site. The PAMs are underlined and SNPs are highlighted in red

Five common single guide RNAs (sgRNAs) neighboring a 5′-NNGRRT-3′ PAM (Supplementary Table S3) were designed, after which the activity of the sgRNA target was measured. The results of electrophoresis postenzyme digestion showed that only sgRNA3 exhibited a high in vitro digestion efficiency, whereas the other sgRNAs showed no digestion activity, which laid the foundation for subsequent gene-knockout experiments (Fig. 4a). sgRNA3 was then constructed into the sgRNA expression vector VK005-101, which was confirmed by sequencing using primer vector-seq-R, and was used for further genetic transformation experiments (Fig. 4b).

### Generation of CRISPR/Cas9-induced *BrOG1* mutations in Chinese cabbage

We cocultivated 200 cotyledon-petiole explants of Chinese cabbage GT-24 via *Agrobacterium tumefaciens* containing a recombinant plasmid. Sixteen resistant buds were obtained after selection, with a differentiation rate of 8%. A total of 16 shoots were then regenerated as rooted plantlets in tissue culture and 9 rooted plantlets were transferred to the greenhouse, of which 6 plantlets survived (named P1 to P6). The T_0_ plants that contained the transgene were selected using Cas_detect_F and CaMVtR primers, which amplified a T-DNA region that contained the CRISPR/Cas9 target sequence and sgRNA (Supplementary Table S4). Five plants (except P3) were identified as transgenic, with a transformation rate of 31.25% based on the number of resistant buds (Fig. 4c). *BrOG*1-com F/R primers flanking the target region were then used to sequence both *BrOG1A* and *BrOG1B* of the transgenic plants (Supplementary Table S4). The sequencing results showed that one plant (P2) had mutations in all the target sequences: two in *BrOG1A* and two in *BrOG1B*. The final transformation rate was 6.25%, based on the number of resistant buds (Fig. 4d). The respective alleles were labeled *A*_2_ and *B*_2_ for a single base A insertion and *A*_3_ and *B*_3_ for a single base T insertion. The P2 plant did not carry the WT (nonmutated) alleles (*A*_1_/*B*_1_) and could be a double heterozygote plant. The CRISPR/Cas9-mediated mutations occurring near the PAM sequence and a single base insertion (A or T) were identified. These frameshift mutations most likely form nonfunctional proteins. Mutations induced by CRISPR/Cas9 causing a single base insertion (A, T, and C) or a two-base deletion (CA) were also found in other knockout lines; however, the nonmutated types (*A*_1_/*B*_1_) were also found (Fig. 4e). Thus, the results indicated the absence of a functional *BrOG1* gene in P2.

### Inheritance of CRISPR/Cas9-induced *BrOG1* mutations

We screened 30 T_1_ plants by PCR using the Cas_detect_F and CaMVtR primers, which revealed 21 transgenic and 9 nontransgenic plants (Fig. 4f), and matched the expected mendelian segregation ratio for a single gene (Supplementary Table S5). This indicated that P2 contained a single Cas9 insertion. Both *BrOG1A* and *BrOG1B* from the 30 T_1_ plants were sequenced. All the plants contained the mutated alleles, suggesting that P2 was a double heterozygote (*A*_2_*A*_3_/*B*_2_*B*_3_). In addition, the segregation pattern was consistent with double gene inheritance and random segregation between genes. The mutations were not associated with the transgene insertion, because we found nontransgenic plants that had all four mutant alleles (such as T2-6 and T2-19). Nine genotypes were obtained, which were expected to have undergone random segregation.

### Off-target analysis

Next, we searched for a possible off-target site of Cas9 endonuclease activities in the P2 mutant plant. We hypothesized that if these activities had occurred, these two mutated sequences would be highly similar to sequences of the *BrOG1* genes (noncoding region on chromosome A02) (Fig. 4g). PCR primer pairs (OffA02F/R) that would bind to the flanking sequences from the potential off-target sites were designed (Supplementary Table S4). We sequenced the PCR products from ten T_1_ offspring and P2 plants. The absence of any sequence variation compared with the Chiifu reference genome indicated that these plants did not contain any off-target mutations in this region. Thus, in one step, we produced a double mutant that did not contain any WT allele but carried mutated *BrOG1A* and *BrOG1B* genes.

### The phenotype of the *BrOG1*-knockout mutants complements that of *BrOG1A*OE mutants

**Fig. 5:**
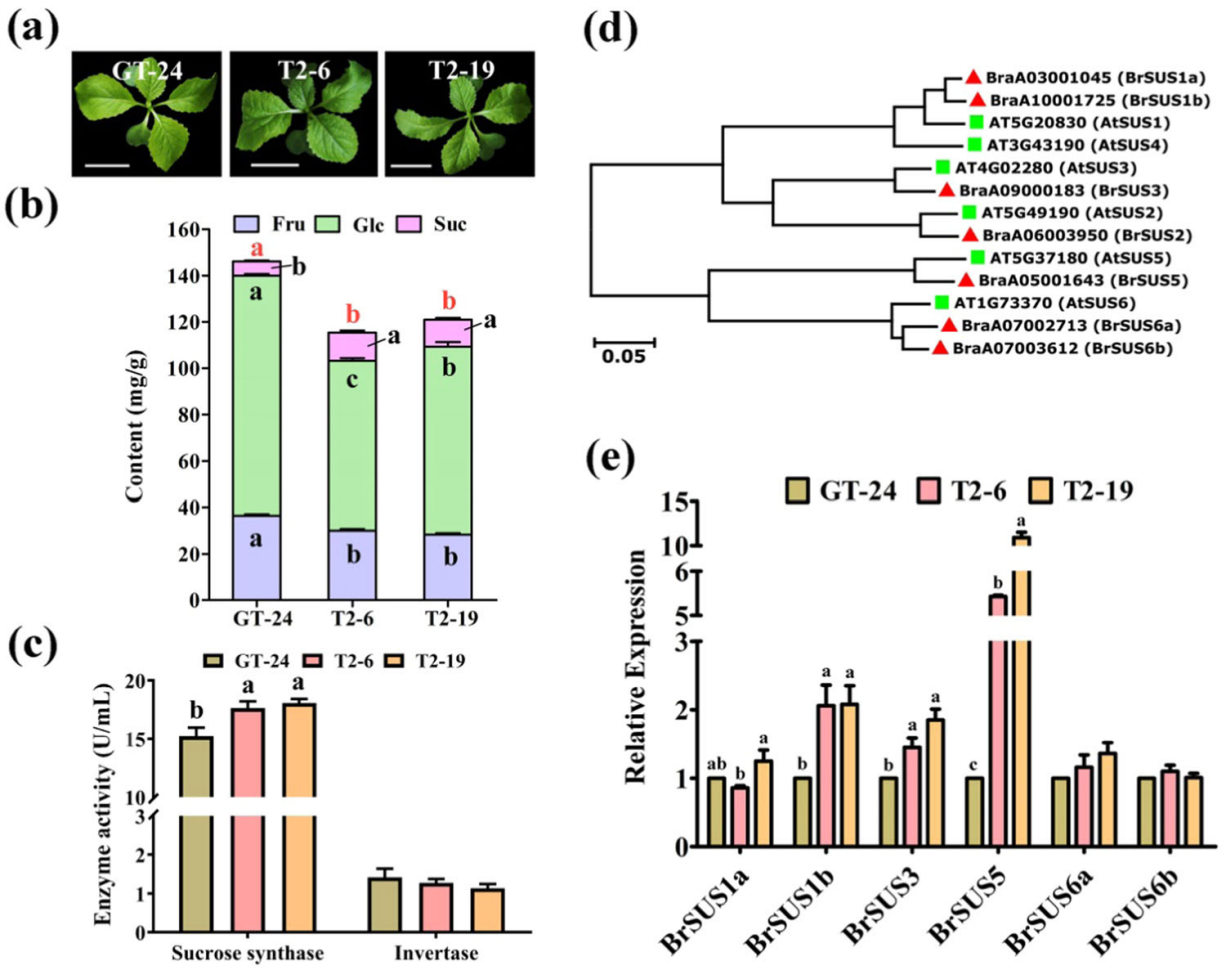
Chinese cabbage *BrOG1* mutants complement the phenotype of *BrOG1A*OE *Arabidopsis* lines. **a** Phenotypes of *BrOG1*-knockout lines. The images were taken upon the appearance of four true leaves (30-day-old plants). Representative T_2_ plants (T2-6 and T2-19) from a T_0_ plant were self-pollinated (P2). GT-24 indicates wild-type plants. The scale bars are 3 cm. **b** Soluble sugar content in 30-day-old wild-type (GT-24) and *BrOG1* mutants. The analysis was performed in triplicate, with fifteen plants per replicate. Fru (fructose, light blue bar), Glc (glucose, light green bar), and Suc (sucrose, light pink bar) were analyzed simultaneously. **c** Detection of the activity of sucrose synthase (SUS) and invertase (INV) using an ELISA. **d** Phylogenetic relationships of SUSs between *A. thaliana* and *B. rapa*. Thirteen amino acid sequences were analyzed. A neighbor-joining tree was constructed by aligning the amino acid sequences. The red solid triangle and green solid box indicate BrSUSs and *AtSUS*s, respectively. **e** Expression levels of *BrSUS*s in GT-24 and *BrOG1* plants. **c**, **e** The analysis was performed in triplicate, with three plants per replicate. GT-24, control plants (light brown bar); T2-6 (light red bar); T2-19 (light orange bar). **b**, **c**, **e** All the values are expressed as the means ± SEs. The black letters indicate significant differences between the mutants and the control (one-way ANOVA, *p* < 0.05). **b** The red letters at the top of the bars indicate significant differences in total soluble sugars between the mutants and the control (one-way ANOVA, *p* < 0.05)

During the seedling stage, we observed no significant phenotypic variations between the *BrOG1*-knockout lines and GT-24 (Fig. 5a). As the overexpression of *BrOG1A* resulted in increased contents of Fru, Glc, and total soluble sugars in *Arabidopsis*, we expected them to decrease in the *BrOG1* knockdown mutants. Similarly, the decrease in Suc content in the *BrOG1A*OE lines should be opposite in the *BrOG1* mutants. We assessed the contents of Fru, Glc, Suc, and total sugars in the T_2_ *BrOG1* mutants and compared them to the contents in GT-24 control plants. We found decreased Fru, Glc, and total sugar contents and increased Suc contents (Fig. 5b). We then compared the activity of BrSUS and BrINV in the mutants with that in the WT to understand the cause behind the variation in soluble sugars. The results showed a higher activity of BrSUS enzymes in *BrOG1* T_2_ lines compared with that in GT-24; however, there was no significant difference in the activity of BrINV (Fig. 5c). We then searched for BrSUSs through protein sequence alignment with six *AtSUS* genes (*AtSUS1–6*) against the *B. rapa* genome through the BLASTP program. A total of seven BrSUSs were identified in the *B. rapa* genome with significant similarity to *AtSUS*s (query cover and identification ≥ 70%) and were labeled BrSUS1a, 1b, 2, 3, 5, 6a, and 6b based on their evolutionary relationship to *AtSUS*s (Fig. 5d). The expression of seven *BrSUS*s was then analyzed via qRT-PCR. Consistent with the increased BrSUS activity in the *BrOG1* mutants, we found elevated expression of *BrSUS1b*, *BrSUS3*, and *BrSUS5* (Fig. 5e). However, there was no significant difference in the expression of *BrSUS6a* and *BrSUS6b* compared with that in the control, and transcripts of *BrSUS2* were not detected. Thus, complementary tests, along with studies on the contents of soluble sugars, enzyme activity, and gene expression in the overexpression and knockout transgenic lines, further substantiated the potential role of *BrOG1* in soluble sugar metabolism.

## Discussion

In this study, we first generated a BrOGOE mutant library in *A. thaliana* and comprehensively investigated the phenotypic variations in the BrOGOE mutants. Based on the importance of primary metabolism in *Brassica* vegetables^12^, we explored the role of the respective mutations in soluble sugar metabolism. Due to the abundance of variation in soluble sugar content in this BrOGOE library, *BrOG1* was randomly chosen for preliminary evaluation of its role in soluble sugar metabolism and CRISPR/Cas9 was used to mediate mutations in *BrOG1* in Chinese cabbage to verify its function.

### Generation of BrOGOE *Arabidopsis* lines

A previous study first investigated the numbers, features, and expression patterns of *OG*s in *B. rapa* (*BrOG*s), providing valuable biological information for exploring the function of *BrOG*s^2^. To explore the functions of these *OG*s, we developed an *A. thaliana* overexpression library using 43 *BrOG*s from *B. rapa* under the control of the CaMV 35S promoter. Functional analysis of *BrOG*s using *A. thaliana* is an efficient method, because it requires relatively little time to analyze gene function. In addition, the floral-dip method is the easiest way to perform highly efficient gene transformation^8,10,28^. These *BrOG*s do not contain any homologous sequences in *A. thaliana* and their ectopic expression would result in phenotypic variations, making it easier to understand the function of *OG*s^2^. Furthermore, *BrOG* function could also be analyzed using *A. thaliana* as a heterologous host, as *OG*s could play similar roles across species^5^. Thus, we speculated that the functions of *BrOG*s in *A. thaliana* might be identical to their roles in *B. rapa*.

The use of the DsRed marker gene under the control of the CaMV 35S promoter increases the convenience of the selection of transgenic seeds through fluorescence^29^. In *A. thaliana*, one or more transgenes are likely to be present in the selected lines, which is typical for the *Agrobacterium*-mediated floral-dip method^11^; thus, we did not investigate the copy number of the T_2_ homozygous mutants. Phenotypic observations of the BrOGOE mutants revealed that only 44% of the mutants displayed visible variations. These results were similar to those of the visual phenotype of the overexpression and RNA interference mutant lines of *Arabidopsis OG QQS*, which are known to be indistinguishable from the WT at all stages—from seedling to senescence^30,31^. In a soybean full-length cDNA overexpression library, a low percentage of mutants exhibited abnormal or favorable phenotypes^10^. Moreover, the overexpression of the *V. unguiculata* OG *UP12_8740* showed no visible phenotype under normal conditions compared with that of the control^4^. Thus, the construction of the *BrOG* mutant library will contribute to revealing the function of OGs.

### BrOGOE in *Arabidopsis* largely influenced soluble sugar metabolism

Chinese cabbage is a popular vegetable, with a long history of cultivation in China. Soluble sugars are essential nutrients and important parameters of vegetable quality^32,33^. The taste characteristics of vegetables can be evaluated based on soluble sugar contents^34^. Therefore, we screened mutants with altered contents of soluble sugars within the BrOGOE mutant library. A high rate of variation in soluble sugar content was observed, which could be due to the unique function of these *BrOG*s. Functional analysis of the *A. thaliana OG QQS* indicated that it could serve as a component of the starch metabolic network in *A. thaliana* leaves, and the increased transcript level of the *QQS* in the sugar-insensitive *sis8* mutant suggests its possible role in maintaining the balance of carbon flow to Suc^31^. Another study showed that *QQS* might integrate primary metabolism and changes in the environment to optimize tolerance to various stresses^35^. Prediction of the cellular roles of *OG*s within the Poaceae indicated that more than 26% of genes were involved in central intermediary and energy metabolism^36^. Many *BrOG*s altered the contents of soluble sugars, suggesting that they play vital roles in the metabolism of soluble sugars; however, the underlying mechanism is unclear and needs further exploration.

### CRISPR/Cas9 caused highly efficient target-gene mutations in Chinese cabbage

Tissue culture in *B. rapa* is challenging; thus, studies on *B. rapa* vegetable transformation are limited, especially with respect to Chinese cabbage^27^. Our laboratory has successfully developed a simple, efficient method for *A. tumefaciens*-induced transformation of Chinese cabbage, in which the ratio of the regenerated plantlets to the total number of cocultivated explants can reach approximately 3%^37^. This efficient transgenic approach promotes breeding and gene functional studies in Chinese cabbage. Recently, compared with traditional mutagenesis strategies, CRISPR/Cas9-mediated mutation has gained popularity as a precise and efficient method for gene functional characterization and crop improvement^21^. Thus, in this study, we used CRISPR/Cas9 to knock out *BrOG1A* and *BrOG1B* in Chinese cabbage. The observed mutation frequency (83.33%) in the T_0_ plants was consistent with that in other plant species, such as *B. napus* and *Oryza sativa*, which suggested that the variations in genome size did not influence the efficiency of the targeted genome editing mediated by CRISPR/Cas9^21,38^.

A recent study reported a high mutation efficiency in genome editing of Chinese cabbage^39^ and the ratio of the regenerated plantlets to the total number of cocultivated explants was relatively low (0.12–1.07%) compared with that in the present study (3%). Based on the T_1_ generation plants, we knew that the mutations were stably inherited regardless of the presence of T-DNA. Similar results were observed for the CRISPR/Cas9-mediated mutations in *B. napus*^21,25^. Frameshift mutations within the common target sequences of *BrOG1A* and *BrOG1B* were induced. The lack of mutations within a potential off-target site with high homology to the sgRNA target sequence suggested that CRISPR/Cas9-induced mutations in Chinese cabbage were highly accurate. Thus, this is the first report on CRISPR/Cas9-mediated mutations of OGs in the Chinese cabbage genome.

### *BrOG1* altered the contents of soluble sugars probably in a SUS-dependent manner

In this study, *BrOG1A*OE was chosen for gene characterization as a representative BrOGOE mutant for functional research. As *BrOG1A* and *BrOG1B* are highly homologous genes (Fig. 3a), *BrOG1A* served as the representative for *BrOG1B* and was overexpressed in *A. thaliana*. We found an insignificant difference between the *BrOG1A*OE mutants and the WT (Fig. 3b and Supplementary Table S2) and a decrease in AtSUS activity and *AtSUS* expression (Fig. 3d, e). Consistent with our findings, *A. thaliana sus1*/*sus2*/*sus3*/*sus4* and *sus5*/*sus6* mutant plants and control plants showed no visible differences, and the levels of AtSUS activity in the leaves and stems were lower in the mutants than in the controls^18,19^. Another study indicated that the loss of SUS activity had minimal influence on root growth under well-aerated conditions^18^. Compared with the control plants, the *BrOG1A*OE plants had high contents of total soluble sugars caused by an increase in the contents of Fru and Glc and lower contents of Suc (Fig. 3c). Multiple *AtSUS* T-DNA insertion lines displayed changes in sugar composition, whereas one mutant did not^20^. Overexpression of *DsSWEET12* (*Dianthus spiculifolius sugars will eventually be exported transporters 12*s) in *A. thaliana* resulted in decreased Suc content; however, the contents of Fru and Glc were higher in the overexpression line than in the WT^40^.

In contrast to these findings in the *BrOG1A*OE mutants, compared with the control plants, the *BrOG1* mutants showed higher levels of BrSUS activity, *BrSUS* expression, and Suc content but lower contents of Fru, Glc, and total soluble sugars. The phenotypes of the *BrOG1A*OE and *BrOG1* mutants and the control plants did not differ, and there was no significant difference in INV activity in the mutants compared with that in the control plants. A previous study showed that most of the functions proposed for INV were unique to specific developmental periods, and that cytosolic INV is indispensable for the normal growth of *A. thaliana* under experimental conditions^18^. Therefore, the phenotypes caused by the knockout of *BrOG1* in Chinese cabbage and the overexpression of *BrOG1A* in *Arabidopsis* were found to be complementary. Moreover, the expression of *AtINV*s or *BrINV*s was not detected, as INVs are regulated at the posttranscriptional level by proteinase inhibitors, kinases, or compartmentalization^41^. The development of large sinks is highly dependent on SUS activity in crop plants, which might be the result of domestication and selection from their wild counterparts^20^, suggesting that *BrOG1* caused variations in the soluble sugar content probably in a SUS-dependent manner in sink cells. These findings indicated that *BrOG*s play vital roles in soluble sugar metabolism and act as important genetic resources for improving the nutritional quality of *B. rapa*.

## Materials and methods

### Plant materials, growth conditions, and characterization of the mutants

*A. thaliana* ecotype Col-0 and the transformed lines were grown under LD conditions (16 h light/8 h dark photoperiod) in the presence of cool-white fluorescence light at 22 ± 1 °C at a relative humidity of 65–70%. For the characterization of BrOGOE mutants, flowering time was measured by counting the number of rosette leaves until flowering or the number of days until the first flower opens, following the methods of a previous study^42^, and rosette radius was determined at the same time by taking the mean of the length of the two largest leaves. The silique length and seed number were measured following a previously described method^43^. Stem height was measured from the central base of the rosette leaves to the main stem top after most siliques turned ripe. Fifteen plants of the BrOGOE lines or Col-0 were randomly investigated.

### Overexpression of the vector constructs, *Arabidopsis* transformation, and selection

Full-length genome sequences of *BrOG*s with 15 bp extensions (BrOGOEc-F/R primers; Supplementary Table S4) were seamlessly inserted into the EcoRI/XhoI linearized plant binary expression vector pBinGlyRed3-35S using a TaKaRa In-Fusion^®^ HD Cloning Kit (Dalian, China, catalog number 639650), following the manufacturer’s instructions. The constructed plasmids were sequenced by Sanger sequencing. The pBinGlyRed3-35S vector was modified from the pBinGlyRed3 vector^29^. The CaMV35Sp-MCS-NOS fragments from the intermediate vector 35S-INT were cloned using a RED3c-F/R primer (Supplementary Table S4) and connected to a BamHI/HindIII linearized pBinGlyRed3 vector using an In-Fusion^®^ HD Cloning Kit, after which the constructed plasmid pBinGlyRed3-35S was sequenced through Sanger sequencing. The DsRed (*Discosoma* red fluorescent protein) marker gene in this vector under the control of the constitutively expressed cauliflower mosaic virus 35S promoter was used to select fluorescent transgenic seeds. The binary vector containing CaMV35Sp-*BrOG*s-NOS was introduced into *A. tumefaciens* GV3101 by the freeze-thaw method. Transgenic *Arabidopsis* Col-0 plants were generated via the floral-dip method^28^.

### Sequence analysis

The Conserved Domain Database (https://www.ncbi.nlm.nih.gov/Structure/cdd/wrpsb.cgi) and the Pfam database (https://pfam.xfam.org/) were used for searching conserved domains. The SignalP Server (http://www.cbs.dtu.dk/services/SignalP/) was used to search signal peptides and protein cleavage sites, and the Plant Transcription Factor Database (PlantTFDB) (http://planttfdb.cbi.pku.edu.cn/) was used to identify transcription factors.

### Construction of the CRISPR/Cas9 vector

Five sgRNAs (Supplementary Table S3) were designed using CRISPR-GE (http://skl.scau.edu.cn/). A SaCas9-gRNA Target Efficiency Detection Kit (ViewSolid Biotech, China, catalog number VK012) was used to determine the sgRNA target efficiency, following the manufacturer’s instructions. The in vitro transcription template of sgRNA included the T7 promoter, target, and sgRNA scaffold sequence. The target efficiency of sgRNA was determined based on the in vitro digestion of the double-stranded DNA of the target gene by sgRNA. The guide sequence was inserted into the gRNA expression vector backbone (VK005-101; Fig. 4b) using annealed oligonucleotides, following the instructions of a SaCas9/gRNA Construction Kit (ViewSolid Biotech, China, catalog number VK005-101). Supplementary Table S4 lists the primers used in this study.

### Chinese cabbage transformation

Chinese cabbage (GT-24, self-propagating material provided by the laboratory) was used for genetic transformation. Seeds of GT-24 were surface sterilized by immersion in 75% ethanol for 30 s and 10% sodium hypochlorite for 1 min, followed by soaking in 2% sodium hypochlorite. The seeds were stirred for 15 min and washed with sterile water (four to five times). Afterward, 10–20 seeds were placed in a glass culture bottle containing 30–40 mL of germination media that consisted of 1/2-strength MS basal media supplemented with 6-BA (5 mg/L), NAA (0.5 mg/L), AgNO_3_ (4 mg/L), Suc (30 g/L), and agar (7 g/L; pH 5.8), and maintained under LD conditions (16 h light/8 h dark photoperiod) in the presence of cool-white fluorescence light at 25 °C. Cotyledons, including petioles (1–2 mm), which were carefully excised from 4-day-old seedlings were used as explants in the transformation experiments. *A. tumefaciens* strain GV3101 containing the working vector was cultured to an OD_600_ of 0.5 in LB liquid media consisting of kanamycin (50 mg/L) and gentamicin (50 mg/L) at 28 °C at 220 r.p.m. The cells were collected and resuspended in DM liquid media (MS basal media with 100 μM acetosyringone) twice. The explants were then suspended in a tenfold diluted infection solution in the DM liquid media for 15 min, with stirring every 5 min. After being blot-dried on sterilized paper towels, the explants were transferred to cocultivation media (MS media supplemented with 6-BA (5 mg/L), NAA (0.5 mg/L), AgNO_3_ (4 mg/L), Suc (30 g/L), agar (7 g/L), and acetosyringone (100 μM) pH 5.8) for 2 days at 25 °C in the dark. The explants were then transferred to shoot induction media (MS media supplemented with 6-BA (5 mg/L), NAA (0.5 mg/L), AgNO_3_ (4 mg/L), Suc (30 g/L), agar (7 g/L), hygromycin (25 mg/L), and timentin (200 mg/L); pH 5.8) until shoot buds developed (~14 days later). Resistant buds excised from the explants were transferred to new shoot induction media. When the hygromycin-resistant shoots were more than 2 cm tall and had more than four leaves, they were transferred to root induction media (MS media supplemented with NAA (0.5 mg/L), IBA (1.5 mg/L), Suc (30 g/L), agar (7 g/L), and timentin (200 mg/L); pH 5.8). The rooted shoots were transplanted into a sterile peat substrate (Pindstrup Horticulture Co., Ltd) and acclimatized in a growth chamber at 25 °C and with 60–70% humidity.

### Detection of mutations induced by CRISPR/Cas9 and off-target analysis

The standard CTAB method was employed to extract DNA from the leaf samples. PCR-based analysis was performed following a previously described method^2^. The target genes were amplified using specific primers (*BrOG*1-com F/R) flanking the designed target sequences. The PCR products were purified using a TaKaRa MiniBEST DNA Fragment Purification Kit (Dalian, China, catalog number 9761) following the manufacturer’s instructions and were cloned into a vector using a TaKaRa pMD™ 18-T Vector Cloning Kit (Dalian, China, catalog number 6011) for further sequencing. A minimum of ten clones from each sample were sequenced through Sanger sequencing. The prediction of the potential off-target sites was performed using CRISPR-GE (http://skl.scau.edu.cn/). Similarly, purified PCR products amplified with OffA02 F/R primers from the samples were cloned into a TaKaRa pMD™ 18-T vector for Sanger sequencing. A minimum of six clones from each sample were sequenced.

### Soluble sugar content and enzyme activity analysis

Preparation of samples for analyzing the soluble sugar content (including Fru, Glc, and Suc) was performed following a previously described method^44^. Lyophilized powder of the aerial tissues collected from 30-day-old BrOGOE, Col-0 plants, *BrOG1* and GT-24 plants (3 biological replicates each, with 15 plants per replicate) was used for extraction. The extraction of soluble sugars was performed using a previously described method^45^ and the sugar contents were analyzed using an UPLC system (Agilent 1290 Infinity II, USA) on an amine column (DiKMA Platisil™ 5 µm NH_2_, 250 × 4.6 mm, China) equipped with a refractive index detector (1290 RID, G7162B). The mobile phase consisted of 75% acetonitrile : 25% water and the flow rate was maintained at 1 mL/min. The temperature of the amine column was maintained at 35 °C. Sample preparation for enzyme activity analysis was identical to the sample preparation for soluble sugar content analysis (three biological replicates with three plants per replicate). The detection of SUS and INV activity was performed using an MLBIO Plant Sucrose Synthase ELISA Kit (catalog number ml077397) and an MLBIO Plant Invertase ELISA Kit (catalog number ml036302), following the manufacturer’s instructions.

### Expression analysis of the *SUS* genes

The sample preparation for the detection of *SUS* gene expression was consistent with the sample preparation for the enzyme activity analysis (three biological replicates with three plants per replicate). Total RNA isolation, cDNA synthesis, and qRT-PCR were performed following previously described methods^2^. The 2^−∆∆Ct^ method^46^ was used to determine gene expression. Supplementary Table S4 lists the primer pairs used.

### Statistical analysis

The *χ*^2^-test was used to determine the confidence level for the segregation ratios of the *BrOG1* T_1_ lines. Other statistical evaluations were performed via Student’s *t*-tests or one-way analysis of variance, followed by individual comparisons with Duncan’s multiple range tests using SPSS software v19.0. Sequence alignment was performed using BioEdit software v7.2.5^47^. Evolutionary relationships of the SUSs in *A. thaliana* and *B. rapa* were inferred using MEGA v6 software^48^. The data were graphically analyzed using GraphPad Prism software v8 (San Diego, CA, USA) and TBtools software v0.665^49^.

## Supplementary information

Figure S1

Table S1

Table S2

Table S3

Table S4

Table S5
