# Supplementary material for: Brassica rapa orphan genes largely affect soluble sugar metabolism
Source: Hortic Res. 2020 Nov 1;7:181. doi: 10.1038/s41438-020-00403-z (PMC7603504; doi:10.1038/s41438-020-00403-z)
Supplement: Supplementary file 1 — Figure S1 [file 41438_2020_403_MOESM1_ESM.pdf]

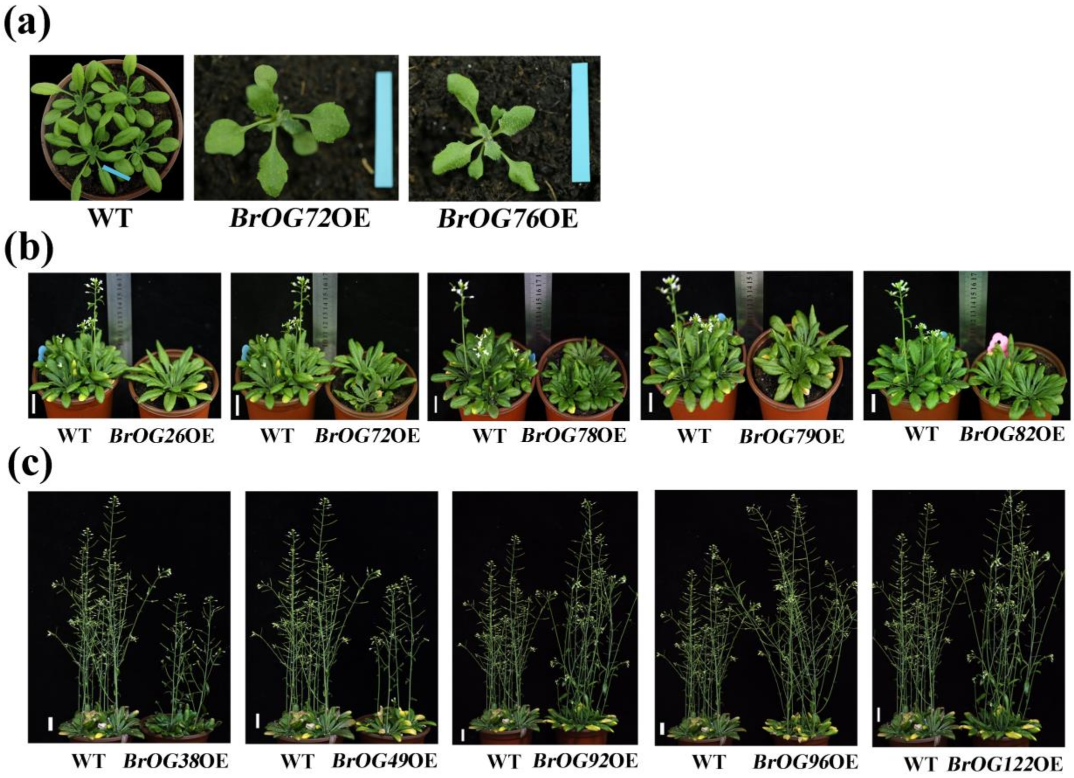

Figure S1 Representative phenotypic variations of BrOGOE mutants. (a) Representative images of 25-day-old BrOGOE mutants and wild type Col-0 (WT). The blue scale bars are 2 cm. (b) Representative images of 37-day-old BrOGOE mutants and wild type Col-0 (WT). The scale bars are 2 cm. (c) Representative images of 47-day-old BrOGOE mutants and wild type Col-0 (WT). The scale bars are 2 cm. All the mutants and WT were grown under LD conditions.
