# Supplementary material for: Brassica rapa orphan genes largely affect soluble sugar metabolism
Source: Hortic Res. 2020 Nov 1;7:181. doi: 10.1038/s41438-020-00403-z (PMC7603504; doi:10.1038/s41438-020-00403-z)
Supplement: Supplementary file 2 — Table S1 [file 41438_2020_403_MOESM2_ESM.pdf]

Table S1 Phenotypic variation data of 43 BrOGOE mutants

Gene name	Gene IDs	Stem height	Rosette radius	Leaf color	Flowering time	Leaf shape	Seed number	Silique length
BrOG1	*BraA08002322*	SHND	RRND	Green	FND	LSND	SNND	SLND
BrOG2	*BraA01002368*	SHND	RRND	Green	FND	LSND	SNND	SLND
BrOG6	*BraA01004777*	SHND	RRND	Green	FND	LSND	SNND	SLND
BrOG10	*BraA02003011*	SHND	RRND	Green	FND	LSND	SNND	SLND
BrOG19	*BraA03003972*	SHND	RRND	Green	FND	LSND	SNND	SLND
BrOG20	*BraA03004165*	SHND	RRND	Green	FND	LSND	SNND	SLND
BrOG23	*BraA03006067*	SHND	RRND	Green	FND	LSND	SNND	SLND
BrOG25	*BraA03006378*	SHND	RRND	Green	FND	LSND	SND	SLD
BrOG26	*BraA04000270*	SHND	RRI	Green	FND	LSND	SNND	SLND
BrOG30	*BraA04000989*	SHND	RRND	Green	FND	LSND	SNND	SLND
BrOG33	*BraA04001243*	SHND	RRND	Green	FND	LSND	SNND	SLND
BrOG36	*BraA04003089*	SHND	RRND	Green	FND	LSND	SNND	SLND
BrOG38	*BraA05001225*	SHD	RRND	Green	FND	LSND	SND	SLND
BrOG40	*BraA05001690*	SHND	RRND	Green	FND	LSND	SNND	SLND
BrOG42	*BraA05001903*	SHND	RRND	Green	FND	LSND	SNND	SLND
BrOG43	*BraA05001928*	SHND	RRND	Green	FND	LSND	SND	SLND
BrOG44	*BraA05002048*	SHND	RRND	Green	FND	LSND	SNND	SLND
BrOG46	*BraA05002203*	SHND	RRND	Green	FND	LSND	SNND	SLND
BrOG49	*BraA05003290*	SHD	RRND	Green	FND	LSND	SNND	SLND
BrOG51	*BraA05004304*	SHND	RRND	Green	FND	LSND	SNND	SLND
BrOG52	*BraA05004654*	SHND	RRND	Green	FND	LSND	SNND	SLND
BrOG53	*BraA06000146*	SHND	RRND	Green	FND	LSND	SND	SLND
BrOG56	*BraA06000317*	SHND	RRND	Green	FND	LSND	SNND	SLND
BrOG59	*BraA06000778*	SHND	RRND	Green	FND	LSND	SNND	SLND
BrOG61	*BraA06001696*	SHD	RRND	Green	FND	LSND	SNND	SLND
BrOG72	*BraA07000650*	SHI	RRD	Green	DF	LSC	SNND	SLND
BrOG76	*BraA07000958*	SHND	RRD	Green	DF	LSC	SND	SLD
BrOG78	*BraA07001082*	SHND	RRD	Green	DF	LSC	SNND	SLND
BrOG79	*BraA07001197*	SHND	RRND	Green	DF	LSC	SNND	SLD
BrOG80	*BraA07001325*	SHND	RRND	Green	FND	LSND	SNND	SLND
BrOG82	*BraA07001442*	SHD	RRND	Green	DF	LSND	SND	SLND
BrOG84	*BraA07001777*	SHND	RRND	Green	FND	LSND	SNND	SLND
BrOG91	*BraA08000338*	SHD	RRND	Green	FND	LSND	SNND	SLND
BrOG92	*BraA08000599*	SHND	RRND	Green	EF	LSC	SNND	SLND
BrOG93	*BraA08000655*	SHND	RRND	Green	FND	LSND	SNND	SLND
BrOG95	*BraA08001348*	SHND	RRND	Green	FND	LSND	SNND	SLND
BrOG96	*BraA08001351*	SHI	RRND	Green	FND	LSND	SNND	SLND
BrOG103	*BraA09001629*	SHND	RRND	Green	FND	LSND	SND	SLD
BrOG111	*BraA09002872*	SHND	RRND	Green	FND	LSND	SNND	SLD
BrOG112	*BraA09003220*	SHND	RRND	Green	FND	LSND	SNND	SLND
BrOG116	*BraA09003463*	SHND	RRND	Green	FND	LSND	SNND	SLND
BrOG122	*BraA09006185*	SHND	RRND	Green	FND	LSND	SND	SLD
BrOG127	*BraA10000785*	SHND	RRND	Yellow	EF	LSND	SNND	SLND
