# Supplementary material for: Brassica rapa orphan genes largely affect soluble sugar metabolism
Source: Hortic Res. 2020 Nov 1;7:181. doi: 10.1038/s41438-020-00403-z (PMC7603504; doi:10.1038/s41438-020-00403-z)
Supplement: Supplementary file 3 — Table S2 [file 41438_2020_403_MOESM3_ESM.pdf]

Table S2 Phenotypic investigation of the *BrOG1A* OE mutants

Phenotypes	WT	*BrOG1A* OE
Flowering Time (d)	32.67 ± 0.68	32.93 ± 0.43
Flowering Time (No. of Leaves)	12.33 ± 0.29	11.67 ± 0.40
Rosette Radius (mm)	53.00 ± 2.88	58.07 ± 1.53
Stem Height (cm)	41.39 ± 1.00	41.01 ± 0.66
Silique Length (mm)	12.87 ± 0.31	12.40 ± 0.29
Seeds/Silique (No.)	50.80 ± 1.49	50.67 ± 1.42

The measurements are the means ± SEs. *, Significantly different values (*, $p < 0.05$, and $p > 0.05$ for those with no marks) according to Student's *t*-tests,
