# Supplementary material for: Brassica rapa orphan genes largely affect soluble sugar metabolism
Source: Hortic Res. 2020 Nov 1;7:181. doi: 10.1038/s41438-020-00403-z (PMC7603504; doi:10.1038/s41438-020-00403-z)
Supplement: Supplementary file 4 — Table S3 [file 41438_2020_403_MOESM4_ESM.pdf]

**Table S3 Summary of the five selected sgRNAs**

| Name | Target site | PAM | GC content of sgRNAs |
|---|---|---|---|
| sgRNA1 | CTCCATTTCGTCTCCAGTTA | TCGAGT | 45% |
| sgRNA2 | CTGATTTTGTATTGTGTTGC | GGGGGT | 35% |
| sgRNA3 | CAAGCCGCCTCTGGTCAATG | GAGAGT | 60% |
| sgRNA4 | AGCTTCATGGTCGTTTCCGT | CGGAGT | 50% |
| sgRNA5 | TTCCGTCGGAGTTATCAGCC | AGGAAT | 55% |
