# Supplementary material for: Brassica rapa orphan genes largely affect soluble sugar metabolism
Source: Hortic Res. 2020 Nov 1;7:181. doi: 10.1038/s41438-020-00403-z (PMC7603504; doi:10.1038/s41438-020-00403-z)
Supplement: Supplementary file 5 — Table S4 [file 41438_2020_403_MOESM5_ESM.pdf]

Table S4 Summary of the primers used in the experiments

Primer name	Sequence (5'→3')	Primer purpose	References
BrOGsOEc-F	GAGAGGACACGAATTC	Vector extensions: over-expression vectors construction of *BrOGs*	Designed in this study
BrOGsOEc-R	TGCGGCCGCCTCGAG		
RED3c-F	ACGCGTAAGGGGATCTCGACGAATTAATTCCAATCCCAC	Vector modification: pBinGlyRed3 modified to pBinGlyRed3-35S	Designed in this study
RED3c-R	ACTCCTCTTAAAGCTTTTAGGTGACACTATAGAATATGCA		
BrOG1-comF	CCCTATATTCCATATGCTATACCCTG	Identification of *BrOG1* gene sequences in GT-24 plants and detection of *BrOG1* mutations	Designed in this study
BrOG1-comR	CCGAAAAATTAAAGAAGCCCTAGAG		
T7-gRNA-FPg	TAATACGACTCACTATAG-**gRNA**-GTTTTAGAGCTAGAAATAGC	Transcription of gRNA *in vitro* for SaCas9-gRNA target efficiency detection	Designed in this study
gRNA-RP	AGCACCGACTCGGTGCCACTT		
BrOG1-sag3-F	TTGCAAGCCGCCTCTGGTCAATG	CRISPR/Cas9 vector construction	Designed in this study
BrOG1-sag3-R	AACCATTGACCAGAGGCGGCTTG		
vector-seq-R	GATGAAGTGGACGGAAGGAAGGAG	Sequencing of constructed CRISPR/Cas9 vector	Designed in this study
Cas_detect_F	AGCCAATACGCAAACCGCCT	Transgene insertion detection of *brog1* mutant	Designed in this study
CaMVtR	CCTCACCAAAATACGAAAGA		
OffA02F	GGGAGCTTCGGCGTGGCTCCT	Off-target analysis	Designed in this study
OffA02R	CCCATCCGATTTCACCTCTTTAGC		
At18sF	GGGCATTCGTATTTCATAGTCAGAG	qRT-PCR analyses of *AtSUSs* expression	Baroja-Fernandez, E. et al. Sucrose synthase activity in the *sus1/sus2/sus3/sus4 Arabidopsis* mutant is sufficient to support normal cellulose and starch production. *Proc. Natl Acad. Sci. USA* **109**, 321-326 (2012).
At18sR	CGGTTCTTGATTAATGAAAACATCCT		
AtSUS1F	AGTTCACTGCGGATATTTTCGC		
AtSUS1R	CCCAACAGTTTCTTTGCTTCCA		
AtSUS2F	TGCCATGAATAATGCCGATTTC		
AtSUS2R	TTGCCCAACATTGTTCTTGCTT		
AtSUS3F	GACCAGACTGATGAGCATGTCG		
AtSUS3R	TCTTCACTTTGTCGAGCCTCG		
AtSUS4F	AAGGAATCGTTCGCAAATGG		
AtSUS4R	TTTCAGCGGCAACATCCTC		
AtSUS5F	GAACGTAGCGTTCGCAGTAA		
AtSUS5R	TGAGATATTGCGTGCTCGAT		
AtSUS6F	CGGAGGCCAGGTTGTTTACAT		
AtSUS6R	AGGCTTGAATCCGAGACCTTGT		
BrEF-1-αF	ATACCAGGCTTGAGCATACCG	qRT-PCR analyses of *BrSUSs* expression	Qi J, Yu S, Zhang F, *et al*. Reference gene selection for real-time quantitative polymerase chain reaction of mRNA transcript levels in Chinese cabbage (*Brassica rapa* L. ssp. *pekinensis*). *Plant Mol. Biol. Rep.* **28**, 597-604 (2010).
BrEF-1-αR	GCCAAAGAGGCCATCAGACAA		
BrSUS1aF	GAAGACCTTGATGTTGAACGAG		Designed in this study
BrSUS1aR	AGAGAAGACGGATCATGTCAAG		
BrSUS1bF	ACATGCTCCGTCTTCTTTTAGA		Designed in this study
BrSUS1bR	CAGAATGTAAACAACCTGACCG		
BrSUS2F	CAGTTCACTGCGGATCTAATTG		Designed in this study
BrSUS2R	TGATTCCGTGAACAACTCTGTA		
BrSUS3F	GCAATGGAAGCCATAGTGATAC		Designed in this study
BrSUS3R	GACTGTTAACTGTTCAACGCTT		
BrSUS5F	ATGAGGATCTTGTCGGAGATTC		Designed in this study
BrSUS5R	GCAAACCAAGAACATCGTTTTG		
BrSUS6aF	GCCGATCAATCTGTTTACTTCC		Designed in this study
BrSUS6aR	CGGTTATGTTCTTCACTGTGTC		
BrSUS6bF	GAAGTGAACGAAACCGAAAAGA		Designed in this study
BrSUS6bR	CTCACTTCTCCTCCAACTGTAG		
