# Supplementary material for: Brassica rapa orphan genes largely affect soluble sugar metabolism
Source: Hortic Res. 2020 Nov 1;7:181. doi: 10.1038/s41438-020-00403-z (PMC7603504; doi:10.1038/s41438-020-00403-z)
Supplement: Supplementary file 6 — Table S5 [file 41438_2020_403_MOESM6_ESM.pdf]

Table S5 Inheritance of CRISPR/Cas9-induced *BrOG1* mutations

Plants	Transgene Genotypes			*brog1* Genotypes									
	Transgenic	Non-transgenic	χ^2 Test[a]	A_2A_2/B_2B_2	A_2A_2/B_2B_3	A_2A_2/B_3B_3	A_2A_3/B_2B_2	A_2A_3/B_2B_3	A_2A_3/B_3B_3	A_3A_3/B_2B_2	A_3A_3/B_2B_3	A_3A_3/B_3B_3	χ^2 Test[b]
O	21	9	0.15	4	3	2	3	11	2	1	3	1	11.33
E	20	10		1.875	3.75	1.875	3.75	7.5	3.75	1.875	3.75	1.875	

[a]3:1 segregation, $\chi^2(0.05, 1) = 3.84$. [b]1:2:1:2:4:2:1:2:1 segregation, $\chi^2(0.05, 8) = 15.51$. O, Observed plant number. E, Expected plant number.
